# Nocturnal Pruritus: The Battle for a Peaceful Night’s Sleep

**DOI:** 10.3390/ijms17030425

**Published:** 2016-03-22

**Authors:** Michael Joseph Lavery, Carolyn Stull, Michael Owen Kinney, Gil Yosipovitch

**Affiliations:** 1 Department of Dermatology/Temple Itch Center, Lewis Katz School of Medicine, Temple University, 3322 North Broad Street-Suite 212, Philadelphia, PA 19140, USA; michael.lavery@tuhs.temple.edu (M.J.L.); tue61103@temple.edu (C.S.); 2 Department of Neurosciences, Royal Victoria Hospital, 274 Grosvenor Road, Belfast, Northern Ireland BT12 6BA, UK; michael.kinney@belfasttrust.hscni.net

**Keywords:** sleep, pruritus, physiology, sleep cycle, circadian rhythm, atopic dermatitis

## Abstract

Chronic pruritus is a debilitating condition with numerous etiologies. Many patients suffer from nocturnal pruritus, which can decrease quality of life and affect mortality in hemodialysis patients. Nocturnal pruritus may occur in all sleep stages but is most prevalent in stages N1 and N2. Further research is needed to elucidate the pathophysiology of nocturnal itch, which will aid in the development of tailored management strategies.

## 1. Introduction

Pruritus, defined as a sensation driving the urge to scratch, may be acute (<6 weeks) or chronic (>6 weeks) [1]. This symptom can significantly impair the quality of life and sleep of affected patients [2,3,4]. Moreover the cumulative effect of such disruptions may influence mortality. The dialysis outcomes and practice patterns (DOPPS) study showed a 17% increased mortality risk among pruritic hemodialysis patients, which was attributed in part to decreased quality of sleep [5].

Sleep is a fundamental restorative neurobiological state that all humans must regularly enter to maintain health. Current sleep duration guidelines recommend 7–9 h per night for individuals 18–64 years old, and 7–8 h for individuals ≥65 years old [6].

However, what drives people to scratch at night—either consciously whereby people are unable to drift off to sleep due to this incessant drive for a further desirable scratch, or unconsciously where people awake in discomfort, to notice areas of excoriations or ulcerated skin? This article will review the physiology of sleep, the different sleep stages disrupted by or associated with nocturnal pruritus and the proposed mechanisms of nocturnal pruritus. In addition, therapeutic options that may reduce this distressing symptom will be discussed.

## 2. The Importance of Sleep

A number of important regulatory functions take place during sleep. Cerebral metabolic waste products are processed, immune surveillance and cytokine regulation is facilitated and processes related to synaptic homeostasis are carried out [7,8,9,10]. Human sleep is very carefully regulated by multiple systems including circadian control [11]. The suprachiasmatic nucleus of the hypothalamus has a fundamental role in synchronizing the 24-h rhythm, which is fundamentally entrained to the light/dark cycle.

Sleep deprivation can have a highly deleterious effect on quality of life. Several adverse effects of sleep insufficiency have been documented, including excessive daytime sleepiness, mood disturbance, and impaired neurocognitive functioning [12]. In addition, insomnia has been shown to negatively impact work or school productivity, increasing both accidents and absenteeism [13]. Sleep deprivation has also been associated with many adverse health outcomes. Several studies have associated lack of sleep with metabolic and endocrine dysregulation. Sleep deprived individuals have been determined to have higher levels of grehlin, which drives appetite, and may lead to weight gain and obesity [14,15]. Such metabolic changes may in turn lead to further complications, such as diabetes mellitus, hypertension, cerebrovascular events and ischemic heart disease. Additionally, lack of sleep has been associated with increased sympathetic nervous system activity, as evidenced by increased blood pressure and cortisol levels [16]. Furthermore, less than adequate sleep has also been associated with impaired immune function, aberrations in judgment, and heightened risk of accidents [11,17].

## 3. Stages of Sleep

Sleep has been divided into two major electrophysiological stages: rapid eye movement (REM) and non-rapid eye movement (NREM) [11]. REM sleep is generated by neurons in the brainstem and NREM sleep is initiated by neurons in the anterior hypothalamus and basal forebrain. Each stage is characterized by distinct electrical features seen on electroencephalogram [11,18] (Table 1).

**Figure 1:**
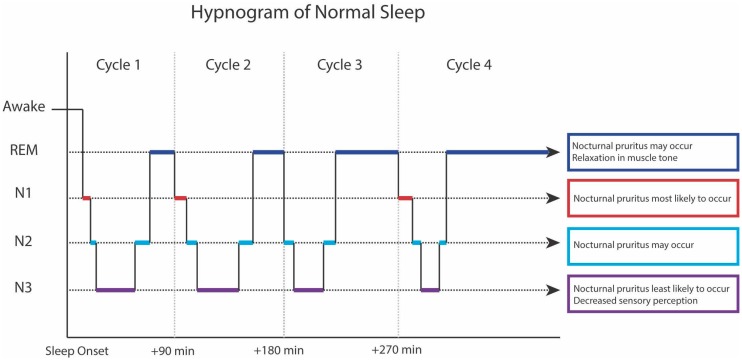
Hypnogram of normal sleep.

NREM sleep is the initial state that one enters from wakefulness. It is subdivided into N1 (formerly stage 1), N2 (formerly stage 2), and N3 (formerly stages 3 and 4). An individual transitions through these stages in a stepwise manner until N3, the deepest stage of NREM sleep, is reached. From N3, a stepwise ascent into REM occurs, completing a sleep cycle (Figure 1). An individual typically transitions through 4–6 cycles per night, each lasting 90–110 min [19]. As sleep progresses over the course of the night, the NREM periods shorten, and the REM periods lengthen. Overall, 20% of sleep is spent in REM, and 80% is spent in NREM [19].

Various polysomnographic (PSG) studies [20,21,22,23] have demonstrated that nocturnal itch can occur in all stages of sleep. In most studies, itch was most prevalent in N1, the lightest sleep stage. Additionally, arousal did not occur prior to itching, but did ensue after scratching behavior. It is also clear that patients with diverse pruritic pathologies experience itch most frequently in N1, N2 and REM and least in stage N3 [21,24]. Deeper, slow wave N3 sleep is associated with reduced sensory perception, which might explain why scratching is less frequent during this stage. Other parameters, such as total wake time, wake time after sleep onset, and the number of nocturnal awakenings were found to be markedly increased in patients with atopic dermatitis (AD). In addition NREM sleep was significantly decreased [24].

Interestingly, results of one PSG study of 14 children with AD indicated that only 15% of arousals in sleep were associated with scratching [25]. This finding suggests that additional factors beyond the mechanistic effect of scratching may trigger arousal. It may be that chronic itch alters the neurochemical milieu, thereby impacting the neural networks involved in sleep maintenance and increasing the frequency of nocturnal arousals. Preliminary research suggests that similar neurotransmitters and some key anatomical locations are involved in both central itch pathways and sleep maintenance pathways [26,27]. Such findings require further exploration and might be amenable to investigation with functional magnetic resonance imaging.

A recent publication of three cases suggested that nocturnal itch may occur in the absence of daytime pruritus and without the presence of a co-existent dermatologic or systemic condition [28]. Such findings suggest that nocturnal pruritus could constitute a parasomnia—abnormal behavior occurring during sleep. Solidifying such a diagnosis would require the use of vigilant and detailed PSG studies.

## 4. Physiological Mechanisms for Nocturnal Pruritus

There have been several proposed pathophysiologic mechanisms underlying nocturnal pruritus. Several key functions of the skin may be altered during sleep, including thermoregulation, maintenance of fluid balance, and barrier function. Aberrations in each of these regulatory mechanisms can contribute to nocturnal pruritus.

Several thermoregulatory variations occur throughout the course of a night. Circadian regulation of sleep dictates that core temperature is maximal in the early evening and minimal in the early morning [11]. Additional temperature variations occur during specific sleep stages. During NREM sleep, the hypothalamic temperature set point is reduced, resulting in dissipation of heat through peripheral vasodilation and increased cutaneous blood flow [29,30]. The consequential increase in skin temperature may be associated with increased itch intensity.

In addition, alteration of skin barrier function occurs during sleep, which may contribute to pruritus. Trans-epidermal water loss (TEWL), a measure of skin barrier integrity, is increased at night [31]. Impairment of barrier function may contribute to itch by facilitating the entry of pruritogens [31]. In patients with AD, increased TEWL has been associated with increased itch intensity [32].

Circadian rhythms may play a role in nocturnal pruritus. The hypothalamus-pituitary-adrenal axis is a complex system that releases corticosteroids through a continuous negative feedback mechanism. Corticosteroids are necessary for mounting an anti-inflammatory reaction and have peak and trough levels commensurate to the time of day. Corticosteroid levels are at a nadir in the evening and night, which leads to a decreased anti-inflammatory response and may facilitate exacerbation of nocturnal pruritus [29]. This corticosteroid level variation has also been associated with nocturnal asthma exacerbation [33].

Disruption of other circadian rhythms may also perpetuate nocturnal itching. Prostaglandin D2 (PGD2) and prostaglandin E2 (PGE2) are elevated at night, and are involved in skin barrier repair after damage from scratching [34]. Patel *et al*. postulate that there is a disruption in this prostaglandin (PG) circadian rhythm in patients suffering from nocturnal pruritus [35]. PGE2 is known to enhance itch and application of topical acetylsalicylic acid, which reduces PGE2, has been shown to alleviate pruritus [36]. Animal studies suggest that PGD2 may have an anti-pruritic effect; however, these results have yet to be replicated in humans [37,38]. In addition, dysregulation of cytokine cascades may have a role in nocturnal pruritus. Several cytokines including IL-2, Il-8 and IL-31 have been related to itch induction and it has been shown that IL-2 levels increase at night [39]. The increase in IL-2 secretion may be secondary to decreased nocturnal levels of cortisol, which acts as an inhibitor of IL-2 production [39]. In contrast interferon-gamma has been shown to decrease itch, with a 50% reduction in pruritus after 1–2 years of treatment in patients with AD. This too may have a diurnal secretion, contributing to nocturnal pruritus [29,40].

Nerve growth factor (NGF), a neurotrophin associated with pruritus, is known to follow a circadian rhythm. One hypothesis suggested that NGF could, therefore, play a role in the pathogenesis of nocturnal pruritus. However, further analysis unexpectedly revealed that both serum and dermal levels of NGF are significantly lower in pruritic AD patients than in healthy controls [41]. Therefore NGF is unlikely to play a significant role in the pathogenesis of nocturnal pruritus.

There are two main opioid receptors involved in the modulation of pruritus—μ-opioid receptors and κ-opioid receptors. Itch may occur after administration of exogenous opioids such as morphine, a μ-opioid receptor agonist. Conversely μ-opioid receptor antagonists and κ-opioid receptor agonists reduce pruritus. It has been suggested that dysregulation in the circadian release of different opioids may play a role in nocturnal pruritus [35].

In addition, hormonal dysregulation could have a role in sleep abnormalities. Melatonin, a regulatory hormone produced by the pineal gland, is involved in maintenance of the sleep cycle and circadian rhythm [42]. One study in children with AD found that lower nocturnal melatonin secretion was significantly associated with sleep disturbance, and that higher nocturnal melatonin secretion was significantly associated with improved sleep quality [24]. Interestingly, the children with AD were found to have higher melatonin levels than healthy controls, suggesting that increased melatonin production may be a compensatory mechanism for sleep impairment.

There may also be a psychological component to nocturnal pruritus. Patients with chronic itch have been shown to have heightened levels of stress and depression. In addition, patients with depression or mood disturbance are known to have disruption of their sleep patterns [29,43]. Decrease in the amount and quality of sleep can contribute to daytime somnolence, irritability, poor concentration and changes in eating habits. In children, AD can have a profound impact on sleep, which may lead to behavioral and neurocognitive deficits. Additionally it has been suggested that nocturnal scratching be included in the list of sleep disruptors that may have an impact on Attention Deficit Hyperactivity Disorder (ADHD) [44].

During the day, minute-to-minute decisions are under higher cortical executive control. In addition our daily activities can distract us from different stimuli, including itch [45]. At night however, the inhibitory action of the executive frontal lobe is reduced which may facilitate the enhancement of nocturnal pruritus. We hypothesize that there may be dysregulation of central mechanisms involved in itch inhibition in patients with nocturnal pruritus. Finally, it has been shown that animals, with severed spinal cords, continue to scratch, thereby suggesting that scratching is a reflex. Concurrently it is possible that there could be a reflexive component to nocturnal scratching [45].

## 5. Conditions that Cause Nocturnal Itch

A myriad of both dermatological and non-dermatological conditions may cause nocturnal pruritus. In clinical practice, patients with chronic pruritus from any cause, commonly complain of nocturnal involvement. Table 2 highlights some of the more common etiologies.

Unpublished data from 150 patients with chronic itch, collected from our institution, shows that 76% of patients suffer from nocturnal pruritus. Conditions with a high prevalence of nocturnal pruritus include advanced age (senile) pruritus, atopic dermatitis, prurigo nodularis, psoriasis, brachioradial pruritus and chronic idiopathic urticaria.

Two major dermatological conditions that cause nocturnal pruritus are AD and psoriasis. Atopic dermatitis is a chronic condition with a prevalence of 7.2% in adults [46] and 10.7% in children [47] in the USA. Clinically AD manifests as pruritic erythematous lesions with associated excoriations, lichenification and/or superimposed infection. AD is characterized by epidermal barrier disruption, which aids the entry of different pruritogens. A complex interaction exists among keratinocytes, cutaneous nerves and immune cells leading to the release of several molecules and cytokines that cause inflammation and pruritus. Chronic pruritus is one of the most common presenting complaints in atopic dermatitis patients with a point prevalence ranging from 87%–100% [48]. In a cross-sectional study of 34,613 adults with AD collated from the National Center for Health Statistics database, Silverberg *et al*. noted increased fatigue and sleep disturbance, as well as decreased sleep efficiency. In addition, patients with AD had more difficulty falling asleep and premature sleep awakening due to pruritus. The combination of atopic dermatitis and sleep disturbance was significantly associated with self-reported fair/poor overall health status [46].

Psoriasis is a chronic inflammatory immune-mediated skin disorder. Prevalence varies greatly by country with a prevalence of 0.91% in the USA and 8.5% in Norway [49]. Activation of the immune system triggers cytokine cascades involved in the different psoriatic cutaneous manifestations. A decrease in core body temperature is important for sleep initiation, and leads to increased heat dissipation and TEWL. However, in psoriasis there is a disorder of heat dissipation and thermoregulation [30,50]. The combination of thermo-dysregulation and an altered itch threshold may have an impact on sleep initiation and quality.

Nocturnal pruritus is commonly associated with infestations, including scabies and bed bugs. The nocturnal pruritus present in scabies can partially be explained by the fact that the mites are more active at night. Moreover the feces (scabella) produced by the mite contain proteases that activate the protease-activated receptor 2 (PAR-2), a known pruritic receptor. Associated infections may cause itch in a similar fashion. In addition the immunological response mounted against the scabies mite (*Sarcoptes scabiei*), activates T-helper 2 (Th2) lymphocytes, releasing several pruritic cytokines including IL-31. Post scabetic pruritus may be caused by perpetuation of the immune response that was mounted against the mite antigen, in combination with central neural hypersensitization. The draft genome of *Sarcoptes scabiei* was recently produced and in the future may usher in both novel investigational and treatment modalities [51].

Bed bugs (*Cimex lenticularis*) are known to cause nocturnal pruritus. They hide around bedboards, picture frames, electric sockets—anywhere that is dark and provides warmth. They are attracted to heat and carbon dioxide (CO_2_). Thus at night they will scurry towards their prey and the exhaled CO_2_ from humans, with victims awakening to papules and excoriations [52]. Recently the entire genome was mapped, identifying genes that are involved in medication resistance [53]. This may pave the way for future targeted gene therapies.

Quality of sleep is poor within the elderly population [54]. In addition chronic pruritus is a common complaint within this age group and patients often complain of nocturnal pruritus. As such, this may be a further compounding factor impacting sleep quality in this population.

Pruritus among patients with psychological diagnoses has been reported. In a cross-sectional study of patients who were being treated in a psychiatric ward, 32% reported suffering from itch, of which 24% felt their itch was worse at night [55]. However, the true percentage of patients suffering from both chronic and nocturnal pruritus is likely to be higher. It is felt that in this cohort of patients there is under-reporting of pruritus, similar to the under-reporting of other symptoms such as pain. In addition some of the medications prescribed for these patients can have anti-pruritic effects and as such psychiatrists may be unintentionally treating pruritus as well as the principal psychological condition [55].

## 6. Sleep Monitoring

Actigraphy is a wrist worn device that measures wrist movement. It emits a piezoelectric bimorph-ceramic cantilevered beam that produces a voltage when the device is moved. It can measure sleep-wake patterns through the frequency and volume of wrist movements [55]. Used alone, it does not require an overnight stay in a sleep lab and is significantly less expensive than other sleep monitoring techniques. Polysomnography is a technique that can record different biophysical changes using several parametric devices including Electoencephalogram (EEG), Electro-oculogram (EOG), Electromyogram (EMG), Electrocardiogram (EKG), pulse oximetry, respiratory and abdominal belts, oronasal pressure sensors and may also include a device to assess snoring levels. Body position is also monitored [18,56]. Both techniques have been used in sleep studies to further evaluate sleep physiology and activity, including scratching [23,57,58,59].

Infra-red videotaping is another method employed for assessment of scratching during sleep. However in a study where both actigraphy and infra-red videotaping were used in a pediatric AD cohort, actigraphy was shown to be equally efficacious in demonstrating scratching behavior. This modality may be preferable for several reasons including its ease of use and low cost [60].

Bender *et al.* [56] utilized PSG and actigraphy in a pilot study of 20 patients with AD to measure disease severity, sleep quality, and scratching. PSG measured sleep latency (time to sleep onset), sleep efficiency (amount of time spent sleeping) and the proportion of time spent in the different sleep stages. Actigraphy also measured sleep latency and sleep efficiency in addition to mean activity, number of wake episodes and longest sleep episode. Increased AD severity and increased nocturnal scratching were associated with decreased sleep efficiency. Scratching activity was increased during the NREM stage of the sleeping cycle. (N2 > N1) [56]. Further studies utilizing PSG and actigraphy to evaluate efficacy of anti-pruritic therapies would be of interest.

Murray *et al.* studied nocturnal pruritus in a cohort of both adult and pediatric patients using Visual Analogue Scale (VAS)—a subjective numeric tool used to evaluate the intensity of pruritus and actigraphy to determine if there was an association between these two reported measurements. Results revealed no significant association between reported VAS scores and actigraphical measurement results, suggesting that frequency of scratching behavior may not be a reliable indicator of perceived itch severity [61]. The development of more accurate applications and devices, with larger storage data, will further advance the assessment of pruritus and sleep.

## 7. Treatments for Nocturnal Pruritus

There are many treatment modalities available from our armamentarium, to treat chronic pruritus. Some of these therapies also improve nocturnal itch. Herein, we discuss several treatment options, which are particularly useful in the management of nocturnal pruritus.

### 7.1. Antihistamines

First-generation antihistamines, including chlorpheniramine, diphenhydramine, hydroxyzine and promethazine, have soporific effects that may provide some relief of nocturnal pruritus [35,62]. Each of these agents antagonizes H_1_ receptors, causing sedation. Hydroxyzine possesses additional antiserotonergic properties, through 5-HT_2A_ (5-hydroxytryptamine 2A) (5-HT_2A_) antagonism, which may aid in the reduction of anxiety.

Second generation antihistamines are generally thought to be of little use in the treatment of nocturnal pruritus, due to their lack of sedative properties. However, in a randomized, placebo-controlled study, oral olopatadine dosed at 5 mg, twice daily, decreased nocturnal scratching in adults with moderate-to-severe AD [63]. Whether other second-generation antihistamines possess similar capabilities is yet to be determined.

### 7.2. Antidepressants

Mirtazapine, an atypical antidepressant that antagonizes noradrenergic (α_1_, α_2_), serotonergic (5-HT_2_, 5-HT_3_), and histaminergic (H_1_) receptors, has demonstrated efficacy in relieving nocturnal itch of varied etiology [64]. Mirtazapine has been reported to reduce nocturnal pruritus in the setting of atopic dermatitis, cholestasis, renal failure, leukemia and lymphoma [65]. While the mechanism underlying mirtazapine’s antipruritic efficacy has not been fully elucidated, it is likely that the anxiolytic effect of 5-HT_2A_ antagonism and the sedative effect of H_1_ antagonism play significant roles. The antipruritic effects of mirtazapine are optimized at nightly doses of 7.5 to 15 mg, below the threshold for antidepressant activity [66]. This agent may be useful as a first line therapy for nocturnal itch, as it is generally well tolerated. In addition, mirtazapine may be used in combination with gabaergic agents for a synergistic effect [38]. The safety profile of mirtazapine is favorable to that of most other antidepressants. Increased appetite and weight gain are among the most frequently reported side effects [65].

Doxepin, a tricyclic antidepressant with antihistaminergic properties, may be useful in the treatment of nocturnal itch. This agent has demonstrated efficacy in reducing pruritus of varied origin, and imparts a sedative effect. Nightly dosages may range from 25 up to 150 mg [67]. Notably, this agent contains anticholinergic properties, which may lead to adverse effects including cardiac abnormalities, blurred vision and dizziness [54]. Therefore, this agent should be used with caution, particularly within the elderly population.

### 7.3. Gabaergics

Structural analogs of gamma-aminobutyric acid (GABA), gabapentin and pregabalin, may be of use in the treatment of nocturnal pruritus. Although their mechanism of action remains unclear, these agents are known to cause sedation. Additionally, they have been shown to reduce pruritus related to neural hypersensitization. This phenomenon plays a role in a variety of pruritic conditions, including neuropathic pruritus and atopic dermatitis. Therefore, these agents may be useful in treating nocturnal itch of varied etiology. In the setting of renal impairment, these agents should be administered at reduced dosages.

### 7.4. κ-Opioid Agonists

Butorphanol, a κ-opioid agonist and partial μ antagonist has demonstrated efficacy in relieving intractable nocturnal pruritus. Administered intranasally at doses ranging from 1 to 4 mg daily, butorphanol has reduced itch and improved sleep in the setting of prurigo nodularis, primary biliary cirrhosis, and lymphoma. Additionally, butorphanol has been shown to be safe and effective in treating nocturnal pruritus associated with advanced age [68]. Butorphanol has a rapid onset of action, and does not cause reversal of analgesia or dependency. This agent is also suitable for patients with hepatic or renal impairment when given at a lower dose.

### 7.5. Benzodiazepines

Benzodiazepines are among the most widely employed treatments for insomnia [69]. This class of sedative agents is thought to increase the proportion of time spent in N2 sleep, and decrease the amount of time spent in N3. However, their efficacy in relieving nocturnal pruritus remains unclear. In a double blind, placebo controlled, crossover study in adults with AD, nitrazepam, dosed at 10 mg nightly, significantly reduced the frequency with which bouts of scratching occurred. However, nitrazepam also lengthened the mean duration of the scratching episodes. Consequently, the total scratching time was not significantly reduced [70]. Benzodiazepines have been associated with many undesirable effects, including tolerance, dependency, and rebound symptoms if discontinued abruptly. Additionally, the half-lives of these agents often exceeds 8 h, thereby increasing the risk of residual fatigue and psychomotor dysfunction [71].

### 7.6. Hormones

Melatonin, a hormone produced by the pineal gland, plays a critical role in sleep regulation and maintenance of circadian rhythm. When administered orally, exogenous melatonin has a sedative effect that may be useful in reducing nocturnal pruritus. In a randomized, double-blind, placebo-controlled trial in children and adolescents with AD, oral melatonin dosed at 3 mg nightly shortened sleep-onset latency by 21.4 min when compared to placebo [72]. No adverse effects were reported throughout the study. Given its favorable safety profile, melatonin may be particularly useful in children with nocturnal pruritus.

### 7.7. Psychological Interventions

Patients who suffer from stress related chronic pruritus, have been shown to benefit from different psychological interventions [73]. These may include relaxation techniques (e.g., progressive muscle relaxation and autogenic training), and habit-reversal training. Other techniques that are useful in patients suffering from chronic pain, may also be useful for chronic pruritus patients and include rational emotive therapy and contextual cognitive behavioural therapy (e.g., mindfulness based stress reduction and acceptance and commitment therapy) [73]. At our institution we have found progressive muscle relaxation to be helpful in reducing nocturnal pruritus in these patients.

## 8. Conclusions

Nocturnal pruritus is common in patients with chronic itch and negatively affects sleep quality. Lack of sleep can have both immediate and long-term effects that lead to medical, social and financial ramifications. As such, a detailed history and comprehensive physical examination is key, to try and minimize these complications. All physicians must be aware of the numerous etiologies of nocturnal pruritus and deliver treatment with a patient-centered approach. Aptly tailored management can help alleviate distress and reduce the complications that result from repetitive scratching. Further studies investigating the pathophysiology of nocturnal pruritus, and its response to treatment are warranted.

## Figures and Tables

**Table 1:** Stages of sleep and the associated features.

Stage of Sleep	Electroencephalogram (EEG) Characteristics and Associated Sleep Stage Features
Wakefulness	Mainly alpha (8–13 Hz) and beta activity (>13 Hz) on EEG
**NREM** Sleep	
N1 (formerly Stage 1) (10% of sleep time)	Slow theta waves (4–7 Hz) emerge on EEG
	Rolling eye movements
	Lightest stage of sleep
N2 (formerly Stage 2) (50% of sleep time)	Background theta wave, less than 20% delta waves. Sleep spindles (central 14–16 Hz rhythms) and K complexes (0.5–1 s high amplitude central sharp waves) on EEG.
	Eye movements usually cease
N3 (formerly Stages 3 + 4) (20% of sleep time)	High amplitude delta waves (<4 Hz) on EEG Also termed slow wave sleep
	Eye movements are slow or absent
	Deepest stage of sleep
**REM** Sleep (20% of sleep time)	Low voltage, mixed frequency background on EEG
	Saccadic eye movements
	80% of dreams occur in this stage. They may be recalled easily especially if immediate awakening after dream onset

(Abbreviations: EEG = electro-encephalogram; EOG = electro-oculogram; NREM = Non-Rapid Eye Movement; REM = Rapid Eye Movement)

**Table 2:** Common causes of nocturnal pruritus.

Category	Disease
Dermatological	Atopic dermatitis
	Psoriasis
	Chronic idiopathic urticaria
	Infestations (scabies, bed bugs, pediculosis, pinworms)
	Lichen planus
	Lichen simplex chronicus
	Prurigo nodularis
Non-Dermatological	Liver disease
	Chronic kidney disease
	Hematopoietic disorders
	Neurological (e.g., brachioradial pruritus)
	Psychological (delusional ideations, depression, schizophrenia, stress)
	Substance abuse
	Advanced age (senile) pruritus
	Restless legs syndrome

## References

[1] Stander S., Weisshaar E., Mettang T., Szepietowski J.C., Carstens E., Ikoma A., Bergasa N.V., Gieler U., Misery L., Wallengren J. (2007). Clinical classification of itch: A position paper of the international forum for the study of itch. Acta Derm. Venereol..

[2] Yosipovitch G., Goon A., Wee J., Chan Y.H., Goh C.L. (2000). The prevalence and clinical characteristics of pruritus among patients with extensive psoriasis. Br. J. Dermatol..

[3] Yosipovitch G., Ansari N., Goon A., Chan Y.H., Goh C.L. (2002). Clinical characteristics of pruritus in chronic idiopathic urticaria. Br. J. Dermatol..

[4] Yosipovitch G., Goon A.T., Wee J., Chan Y.H., Zucker I., Goh C.L. (2002). Itch characteristics in Chinese patients with atopic dermatitis using a new questionnaire for the assessment of pruritus. Int. J. Dermatol..

[5] Pisoni R.L., Wikström B., Elder S.J., Akizawa T., Asano Y., Keen M.L., Saran R., Mendelssohn D.C., Young E.W., Port F.K. (2006). Pruritus in haemodialysis patients: International results from the dialysis outcomes and practice patterns study (DOPPS). Nephrol. Dial. Transplant..

[6] Hirshkowitz M., Whiton K., Albert S.M., Alessi C., Bruni O., DonCarlos L., Hazen N., Herman J., Hillard P.J., Katz E.S. (2015). National Sleep Foundation’s updated sleep duration recommendations: Final report. Sleep Health.

[7] Ruiz F.S., Andersen M.L., Martins R.C., Zager A., Lopes J.D., Tufik S. (2012). Immune alterations after selective rapid eye movement or total sleep deprivation in healthy male volunteers. Innate Immun..

[8] Porkka-Heiskanen T. (2013). Sleep homeostasis. Curr. Opin. Neurobiol..

[9] Imeri L., Opp M.R. (2009). How (and why) the immune system makes us sleep. Nat. Rev. Neurosci..

[10] Irwin M.R. (2015). Why sleep is important for health: A psychoneuroimmunology perspective. Annu. Rev. Psychol..

[11] Benca R. (2012). Sleep Disorders: The Clinician’s Guide to Diagnosis and Management.

[12] Dinges D.F., Pack F., Williams K., Gillen K.A., Powell J.W., Ott G.E., Aptowicz C., Pack A.I. (1997). Cumulative sleepiness, mood disturbance, and psychomotor vigilance performance decrements during a week of sleep restricted to 4–5 hours per night. Sleep.

[13] Daley M., Morin C.M., LeBlanc M., Gregoire J.P., Savard J., Baillargeon L. (2009). Insomnia and its relationship to health-care utilization, work absenteeism, productivity and accidents. Sleep Med..

[14] Spiegel K., Leproult R., van Cauter E. (1999). Impact of sleep debt on metabolic and endocrine function. Lancet.

[15] Spiegel K., Tasali E., Penev P., van Cauter E. (2004). Brief communication: Sleep curtailment in healthy young men is associated with decreased leptin levels, elevated ghrelin levels, and increased hunger and appetite. Ann. Intern. Med..

[16] Tochikubo O., Ikeda A., Miyajima E., Ishii M. (1996). Effects of insufficient sleep on blood pressure monitored by a new multibiomedical recorder. Hypertension.

[17] Watson N.F., Badr M.S., Belenky G., Bliwise D.L., Buxton O.M., Buysse D., Dinges D.F., Gangwisch J., Grandner M.A., Kushida C. (2015). Recommended amount of sleep for a healthy adult: A joint consensus statement of the American Academy of Sleep Medicine and Sleep Research Society. Sleep.

[18] Clarke C., Howard R., Rossor M., Shorvon S. (2009). Neurology: A Queen Square Textbook.

[19] Chokroverty S. (2010). Overview of sleep & sleep disorders. Indian J. Med. Res..

[20] Aoki T., Kushimoto H., Hishikawa Y., Savin J.A. (1991). Nocturnal scratching and its relationship to the disturbed sleep of itchy subjects. Clin. Exp. Dermatol..

[21] Savin J.A., Paterson W.D., Oswald I., Adam K. (1975). Further studies of scratching during sleep. Br. J. Dermatol..

[22] Monti J.M., Vignale R., Monti D. (1989). Sleep and nighttime pruritus in children with atopic dermatitis. Sleep.

[23] Koca R., Altin R., Konuk N., Altinyazar H.C., Kart L. (2006). Sleep disturbance in patients with lichen simplex chronicus and its relationship to nocturnal scratching: A case control study. South Med. J..

[24] Chang Y.S., Chou Y.T., Lee J.H., Lee P.L., Dai Y.S., Sun C., Lin Y.T., Wang L.C., Yu H.H., Yang Y.H. (2014). Atopic Dermatitis, melatonin and sleep disturbance. Pediatrics.

[25] Reuveni H., Chapnick G., Tal A., Tarasiuk A. (1999). Sleep fragmentation in children with atopic dermatitis. Arch. Pediatr. Adolesc. Med..

[26] Ikoma A., Steinhoff M., Ständer S., Yosipovitch G., Schmelz M. (2006). The neurobiology of itch. Nat. Rev. Neurosci..

[27] Dhand A., Aminoff M.J. (2014). The neurology of itch. Brain.

[28] Nigam G., Riaz M., Hershner S.D., Goldstein C.A., Chervin R.D. (2016). Sleep Related Scratching: A Distinct Parasomnia?. J. Clin. Sleep Med..

[29] Thornburn P.T., Riha R.L. (2010). Skin disorder and sleep in adults: Where is the evidence?. Sleep Med. Rev..

[30] Gupta M.A., Simpson F.C., Gupta A.K. (2016). Psoriasis and sleep disorders: A systematic review. Sleep Med. Rev..

[31] Yosipovitch G., Xiong G.L., Haus E., Sackett-Lundeen L., Ashkenazi I., Maibach H.I. (1998). Time-dependent variations of the skin barrier function in humans: Transepidermal water loss, stratum corneum hydration, skin surface pH, and skin temperature. J. Investig. Dermatol.

[32] Lee C.H., Chuang H.Y., Shih C.C., Jong S.B., Chang C.H., Yu H.S. (2006). Transepidermal water loss, serum IgE and β endorphin as important and independent biological markers for development of itch intesnity in atopic dermatitis. Br. J. Dermatol..

[33] Smolensky M.H., Reinberg A.E., Martin R.J., Haus E. (1999). Clinical chronobiology and chronotherapeutics with applications to asthma. Chronobiol. Int..

[34] Honma Y., Arai I., Hashimoto Y., Futaki N., Sugimoto M., Tanaka M., Nakaike S. (2005). Prostaglandin D2 and prostaglandin E3 accelerate the recovery of cutaneous barrier disruption induced by mechanical scratching in mice. Eur. J. Pharmacol..

[35] Patel T., Ishiuji Y., Yosipovitch G. (2007). Nocturnal itch: Why do we itch at night?. Acta Derm. Venereol..

[36] Leslie T.A., Greaves M.W., Yosipovitch G. (2015). Current topical and systemic therapies for itch. Handb. Exp. Pharmacol..

[37] Sugimoto M., Arai I., Futaki N., Hashimoto Y., Honma Y., Nakaike S. (2006). COX-1 inhibition enhances scratching behavior in NC/Nga mice with atopic dermatitis. Exp. Dermatol..

[38] Stull C., Lavery M.J., Yosipovitch G. (2015). Advances in therapeutic strategies for the treatment of pruritus. Expert Opin. Pharmacother..

[39] Lissoni P., Rovelli F., Brivio F., Brivio O., Fumagalli L. (1998). Circadian secretions of IL-2, IL-12, IL-6 and IL-10 in relation to the light/dark rhythm of the pineal hormone melatonin in healthy humans. Nat. Immun..

[40] Steinhoff M., Bienenstock J., Schmelz M., Maurer M., Wei E., Bíró T. (2006). Neurophysiological, neuroimmunological, and neuroendocrine basis of pruritus. J. Investig. Dermatol..

[41] Papoiu A.D.P., Wang H., Nattkemper L., Tey H.L., Ishiuji Y., Chan Y.H., Schmelz M., Yosipovitch G. (2011). A study of serum concentrations and dermal levels of NGF in atopic dermatitis and healthy subjects. Neuropeptides.

[42] Schwarz W., Birau N., Hornstein O.P., Heubeck B., Schönberger A., Meyer C., Gottschalk J. (1988). Alterations of melatonin secretion in atopic eczema. Acta Derm. Venereol..

[43] Gelder M., Mayou R., Geddes J. (2005). Mood disorders. Psychiatry.

[44] Camfferman D., Kennedy J.D., Gold M. (2010). Eczema and sleep and its relationship to daytime functioning in children. Sleep Med. Rev..

[45] Sack R., Hanifin J. (2010). Scratching below the surface of sleep and itch. Sleep Med. Rev..

[46] Silverberg J.I., Garg N.K., Paller A.S., Fishbein A.B., Zee P.C. (2015). Sleep disturbances in adults with eczema are associated with impaired overall health: A US population-based study. J. Investig. Dermatol..

[47] Shaw T.E., Currie G.P., Koudelka C.W., Simpson E.L. (2011). Eczema prevalence in the United States: Data from the 2003 national survey of children’s health. J. Investig. Dermatol..

[48] Mollanazar N.K., Smith P.K., Yosipovitch G. (2015). Mediators of chronic pruritus in atopic dermatitis: Getting the itch out?. Clin. Rev. Allergy Immunol..

[49] Parisi R., Symmons D.P.M., Griffiths C.E.M., Ashcroft D.M. (2013). Global epidemiology of psoriasis: A systematic review of incidence and prevalence. J. Investig. Dermatol..

[50] Leibowitz E., Seidman D.S., Laor A., Shapiro Y., Epstein Y. (1991). Are psoriatic patients at risk of heat intolerance?. Br. J. Dermatol..

[51] Rider S.D., Morgan M.S., Arlian L.G. (2015). Draft genome of the scabies mite. Parsit. Vectors.

[52] Lavery M.J., Parish L.C. (2011). Bed bugs revisited. Skinmed.

[53] Rosenfeld J.A., Reeves D., Brugler M.R., Narechania A., Simon S., Durrett R., Foox J., Shianna K., Schatz M.C., Gandara J. (2016). Genome assemnly and geospatial phylogenomics of the bed bug Cimex lectularius. Nat. Commun..

[54] Valdes-Rodriguez R., Stull C., Yosipovitch G. (2015). Chronic pruritus in the elderly: Pathophysiology, diagnosis and management. Drugs Aging.

[55] Mazeh D., Melamed Y., Cholostoy A., Aharonovitzch V., Weizman A., Yosipovitch G. (2008). Itching in the psychiatric ward. Acta Derm. Venereol..

[56] Bender B.G., Ballard R., Canono B., Murphy J.R., Leung D.Y. (2008). Disease severity, scratching, and sleep quality in patients with atopic dermatitis. J. Am. Acad. Dermatol..

[57] Bender B.G., Leung S.B., Leung D.Y. (2003). Actigraphy assessment of sleep disturbance in patients with atopic dermatitis: An objective life quality measure. J. Allergy Clin. Immunol..

[58] Bringhurst C., Waterston K., Schofield O., Benjamin K., Rees J.L. (2004). Measurement of itch using actigraphy in pediatric and adult populations. J. Am. Acad. Dermatol..

[59] Ebata T., Iwasaki S., Kamide R., Niimura M. (2001). Use of a wrist activity monitor for the measurement of nocturnal scratching in patients with atopic dermatitis. Br. J. Dermatol..

[60] Benjamin K., Waterston K., Russell M., Schofield O., Diffey B., Rees J.L. (2004). The development of an objective method for measuring scratch in children with atopic dermatitis suitable for clinical use. J. Am. Acad. Dermatol..

[61] Murray C.S., Rees J.L. (2011). Are subjective accounts of itch to be relied on? The lack of relation between visual analogue itch scores and actigraphic measures of scratch. Acta Derm. Venereol..

[62] Patel T., Yosipovitch G. (2010). Therapy of pruritus. Expert Opin. Pharmacother..

[63] Yamanaka K., Motomura E., Noro Y., Umeda K., Morikawa T., Umeda-Togami K., Omoto Y., Isoda K., Kondo M., Tsuda K. (2015). Olopatadine, a non-sedating H1 antihistamine, decreases the nocturnal scratching without affecting sleep quality in atopic dermatitis. Exp. Dermatol..

[64] Hundley J.L., Yosipovitch G. (2004). Mirtazapine for reducing nocturnal itch in patients with chronic pruritus: A pilot study. J. Am. Acad. Dermatol..

[65] Davis M.P., Frandsen J.L., Walsh D., Andresen S., Taylor S. (2003). Mirtazapine for pruritus. J. Pain Symptom Manag..

[66] Puzantian T. (1998). Mirtazapine, an antidepressant. Am. J. Health Syst. Pharm..

[67] Yosipovitch G., Samuel L.S. (2008). Neuropathic and psychogenic itch. Dermatol. Ther..

[68] Dawn A.G., Yosipovitch G. (2006). Butorphanol for treatment of intractable pruritus. J. Am. Acad. Dermatol..

[69] Roux F.J., Kryger M.H. (2010). Medication effects on sleep. Clin. Chest Med..

[70] Ebata T., Izumi H., Aizawa H., Kamide R., Niimura M. (1998). Effects of nitrazepam on nocturnal scratching in adults with atopic dermatitis: A double-blind placebo-controlled crossover study. Br. J. Dermatol..

[71] Asnis G.M., Thomas M., Henderson M.A. (2016). Pharmacotherapy treatment options for insomnia: A primer for clinicians. Int. J. Mol. Sci..

[72] Chang Y.S., Lin M.H., Lee J.H., Lee P.L., Dai Y.S., Chu K.H., Sun C., Lin Y.T., Wang L.C., Yu H.H. (2016). Melatonin supplementation for children with atopic dermatitis and sleep disturbance: A randomized clinical trial. JAMA Pediatr..

[73] Schut C., Mollanazar N.K., Kupfer J., Gieler U., Yosipovitch G. (2016). Psychological interventions in the treatment of chronic itch. Acta Derm. Venereol..

